# The impact of robotic intervention on joint attention in children with autism spectrum disorders

**DOI:** 10.1186/s13229-018-0230-8

**Published:** 2018-09-04

**Authors:** Hirokazu Kumazaki, Yuichiro Yoshikawa, Yuko Yoshimura, Takashi Ikeda, Chiaki Hasegawa, Daisuke N. Saito, Sara Tomiyama, Kyung-min An, Jiro Shimaya, Hiroshi Ishiguro, Yoshio Matsumoto, Yoshio Minabe, Mitsuru Kikuchi

**Affiliations:** 1 Research Center for Child Mental Development, Kanazawa University, 13-1, Takaramachi, Kanazawa, Ishikawa 920-8640 Japan; 2 Department of Systems Innovation, Graduate School of Engineering Science, Osaka University, 1-3, Machikaneryamachou, Toyonaka, Osaka, 560-0043 Japan; 3 Service Robotics Research Group, Intelligent Systems Institute, National Institute of Advanced Industrial Science and Technology, Ibaraki, 305-8560 Japan

**Keywords:** Autism spectrum disorders, Typical development, Intervention, Joint attention, Robot

## Abstract

**Background:**

A growing body of anecdotal evidence indicates that the use of robots may provide unique opportunities for assisting children with autism spectrum disorders (ASD). However, previous studies investigating the effects of interventions using robots on joint attention (JA) in children with ASD have shown insufficient results. The robots used in these studies could not turn their eyes, which was a limitation preventing the robot from resembling a human agent.

**Methods:**

We compared the behavior of children with ASD with that of children with typical development (TD) during a JA elicitation task while the children interacted with either a human or a robotic agent. We used the robot “CommU,” which has clear eyes and can turn its eyes, for the robotic intervention. The age range of the participants was limited to 5–6 years.

**Results:**

Sixty-eight participants participated in this study, including 30 (10 females and 20 males) children with ASD and 38 (13 females and 25 males) children with TD. The participants were randomly assigned to one of the following two groups: the robotic intervention group or the control group. JA in the children with ASD was better during the robotic intervention than during the human agent intervention. These children exhibited improved performance in the JA task with human after interacting with the robot CommU. JA was differentially facilitated by the human and robotic agents between the ASD and TD children.

**Conclusions:**

The findings of this study significantly contribute to the literature on the impact of robots on JA and provide information regarding the suitability of specific robot types for therapeutic use.

**Electronic supplementary material:**

The online version of this article (10.1186/s13229-018-0230-8) contains supplementary material, which is available to authorized users.

## Background

Autism spectrum disorders (ASD) are characterized by social communication deficits and a tendency to engage in repetitive behaviors [1]. A core social-communication deficit observed in children with ASD is limited joint attention (JA) behaviors. JA refers to a social exchange in which a child coordinates attention with a social partner or aspect of the environment by the acts of eye-gazing and pointing or other verbal or non-verbal indications. JA serves as a foundation for developing communicative competence and early social and cognitive skills [2–9]. Early interventions that facilitate JA are promising because these strategies increase children’s opportunities to learn from their environment and change their developmental trajectories [10, 11].

To engage in JA, children must orient toward their social partners and shift attention rapidly between social and non-social stimuli in their surroundings [12, 13]. Children with ASD require a well-suited interaction partner to develop JA skill [14]. In many cases, children with ASD do not show sustained motivation to interact with an interaction partner. For caregivers and trainers, concentrating on interactions with children with ASD is a difficult task [15, 16].

Children with ASD preferentially orient visually toward non-social objects, such as robots, rather than social objects [17–19]. These children prefer non-social objects because they are predictable, simple, and easy to comprehend. The use of robots may provide unique opportunities for assisting children with ASD [20–25]. For example, children with ASD exhibit improved performance in imitation tasks using a robot [22, 26]. However, previous studies [14, 27, 28] investigating the efficacy of interventions using robots on JA in children with ASD have shown insufficient results because these studies used a robot (i.e., “Nao”) that cannot turn its eyes. The robot’s inability to turn its eyes was a limitation that prevented the robot from resembling a human agent, and eye-gazing is among the primary elements of JA [28, 29].

**Fig. 1:**
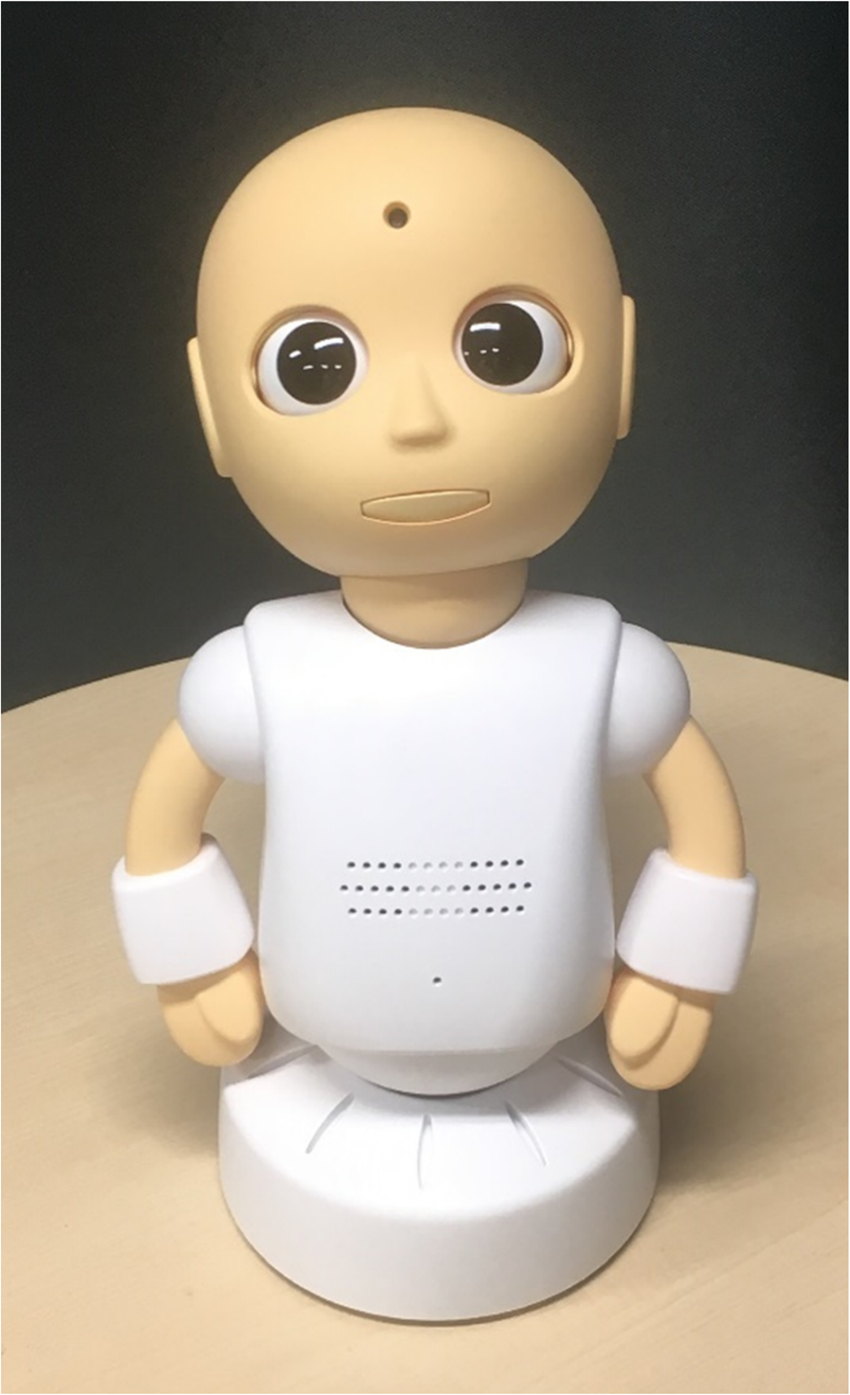
CommU

Thus, we selected the communication robot “CommU” (Fig. 1; Vstone Co., Ltd.) [30, 31] to facilitate JA. CommU has clear eyes and can turn its eyes. Because eye contact is a basic social skill that children with ASD often lack, CommU’s clear eyes allow the children to recognize and interpret the communication signals and are expected to facilitate JA.

We compared the behavior of children with ASD with that of children with typical development (TD) during a JA elicitation task while the children interacted with a human or a robotic agent. The primary objective of this study was to test whether the robot is more useful in facilitating JA than a human agent in children with ASD during an interactive session. Second, we tested whether children with ASD show improvement in JA during human interactions after interacting with the robot CommU. Third, we tested whether the robot is more useful in facilitating JA in children with ASD than children with TD. We hypothesized that (a) children with ASD would demonstrate better JA under the CommU condition than under the human agent condition, (b) children with ASD would show improvement in JA tasks with a human after interacting with CommU, and (c) the facilitative effect of JA due to robot intervention would be larger in children with ASD than in TD children.

The age of the participants naturally affects the outcome of experiments investigating JA. JA performance in older children with ASD (mean age 9.25 ± 1.87 years) is similar to that in TD children during interactions with a human agent [14]. However, these experiments are too difficult for younger children to complete. In fact, in a previous study [28] involving children with ASD under 5 years of age, many participants dropped out of the study. In our preliminary study (unpublished), many children younger than 4 years of age were afraid of CommU and could not participate in the study. This confounding factor should be minimized using subjects within a narrow age range over 5 years. In addition, Vailouli et al. [32] suggested that challenges with JA do not abate, even at the time the child enters elementary school. There are several studies reporting the efficacy of JA intervention in children with ASD older than 5 years of age. For example, Vailouli et al. [32] have shown that JA intervention for children with ASD between the ages of 5 and 7 years was effective in promoting social engagement. Eissa [33] showed that JA intervention for children with ASD between the ages of 5 and 7 years was effective in improving eye contact, gesturing, following instructions, initiating caressing/singing, and communication skills. Therefore, we studied participants whose age range was limited to 5–6 years.

## Methods

### Participants

The present study was approved by the ethics committee of Kanazawa University. All participants were recruited from the Research Center for Child Mental Development, Kanazawa University. All procedures involving human participants were conducted according to the ethical standards of the institutional and/or national research committee and the 1964 Helsinki Declaration and its subsequent amendments or comparable ethical standards. After providing a complete explanation of the study, all participants provided written informed consent. All participants and their guardians agreed to participate in the study. The inclusion criteria for the participants were as follows: (1) age 5–6 years, (2) mental processing score on the Kaufman Assessment Battery for Children (K-ABC) [34] ≥ 70, and (3) acquisition score on the K-ABC ≥ 70. The K-ABC was employed to estimate the intelligence levels of the children. The children with ASD were diagnosed using the Autism Diagnostic Observational Schedule-Generic (ADOS-G) [35], the Diagnostic Interview for Social and Communication Disorders (DISCO) [36], and the DSM-5 criteria at the time of recruitment for this study. Children with ASD were included in this study if they met the diagnosis criteria for childhood autism, atypical autism or Asperger’s syndrome with DISCO or the ADOS criteria for an autism spectrum disorder.

The parents of the children in the TD group completed the Social Communication Questionnaire (SCQ) [37] to screen for clinically significant ASD symptoms in the TD children. Furthermore, to exclude children with psychiatric diagnoses, the Mini-International Neuropsychiatric Interview for Children and Adolescents (MINI Kids) [38, 39] was administered.

### Procedures

**Fig. 2:**
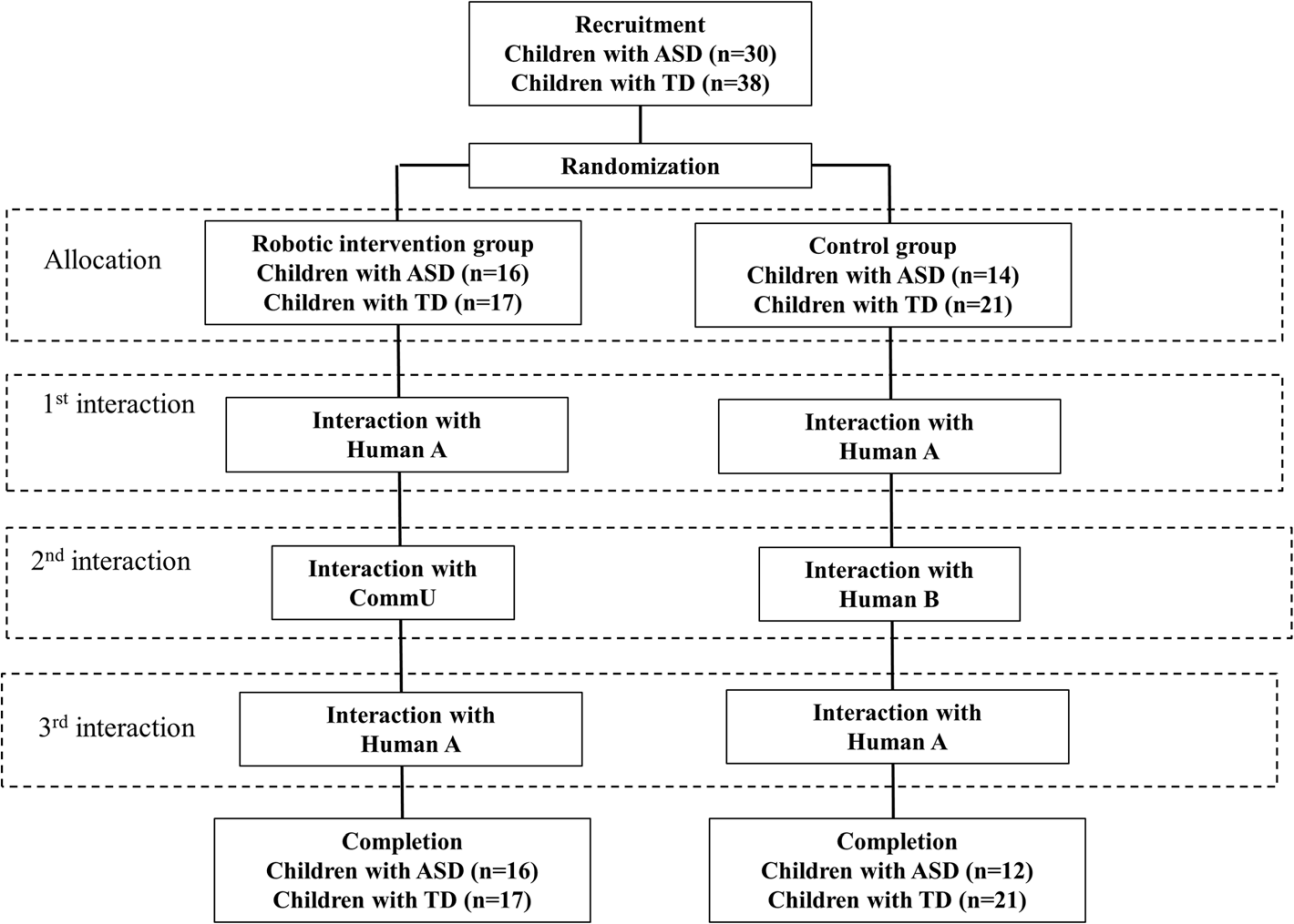
Participant flow

Both the children with ASD and the TD children were randomly assigned to one of two groups (see Fig. 2). The participants completed a sequence of three interaction conditions that were done consecutively within the same visit. In the robotic intervention group, the participants interacted with “human A,” “CommU,” and “human A.” In the control group, the participants interacted with “human A,” “human B,” and “human A.” The participants were informed of the interaction order after the group assignments. During each session, the participants interacted with the robot or human agent for approximately 5 min (i.e., the participants in each group had approximately 15 min of total interaction). During each interaction, a human agent or “CommU” followed a specific interview script and protocol. Across the sessions, the scripts were slightly varied to promote engagement but followed the same basic structure. Please refer to the Additional file 1 for examples of the scripts.

During the “CommU” session, the robot was placed on a table in the middle of the room. To elicit the belief that the robot behaved and responded autonomously, we adopted a Wizard-of-Oz scheme similar to the systems conventionally used in robotics studies [40]. Specifically, the robot was operated by the researchers, who sat in front of a terminal computer located against a wall in the experimental room; the researchers were not visible during the trial. The participants were not informed that the robots were controlled by the researchers. The researchers operated the robots according to the prepared scripts.

**Fig. 3:**
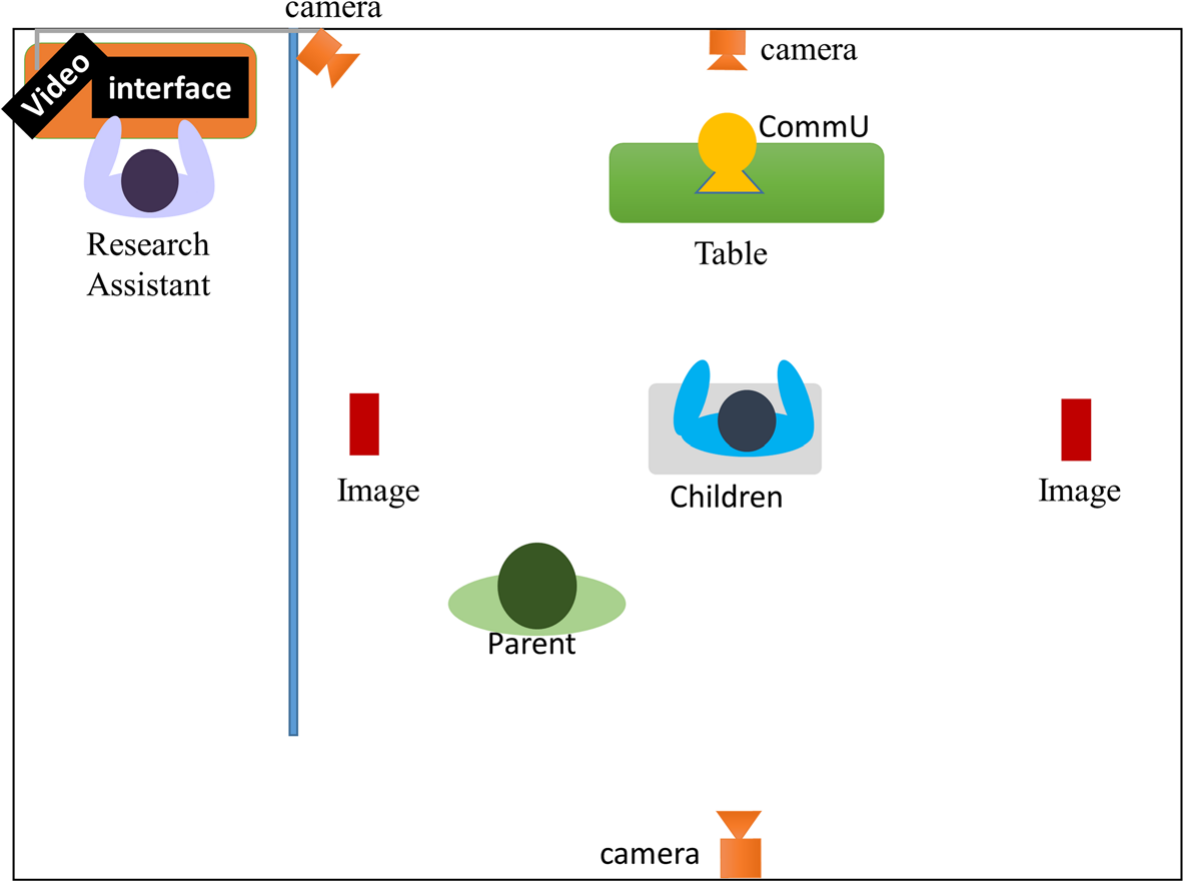
Experimental room setting during the robotic intervention session

**Fig. 4:**
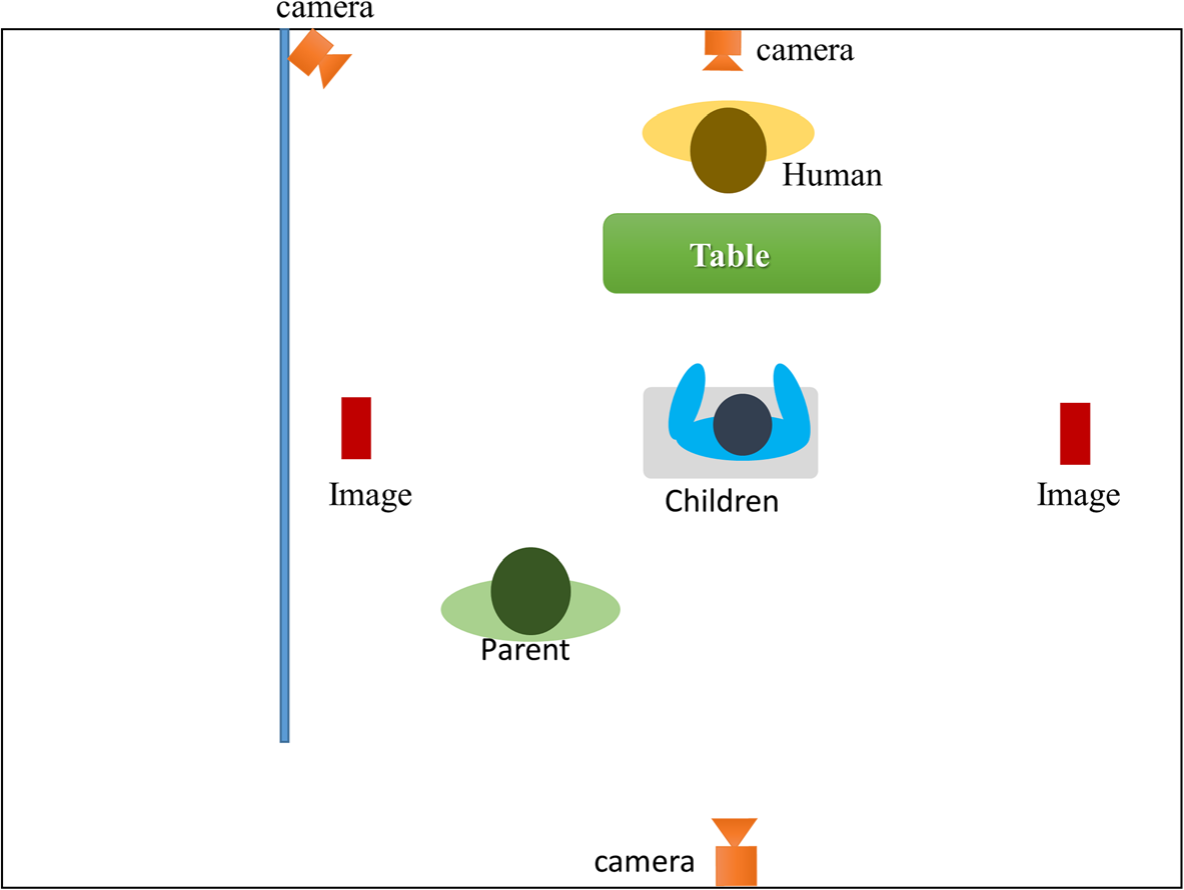
Experimental room setting during the human agent session

To capture the relevant information, a simple joint interaction performance was prepared. A child-sized table with chairs was set up in the middle of the experimental room. The parents were invited to sit 150 cm diagonally behind their child. CommU or a human agent was seated in front of the participant at a distance of 150 cm. We placed CommU on a desk at a height similar to that of a human agent. Two images were placed on the left and right sides of the participant, which were used as the foci of attention by the system. The images were 21 cm × 29.7 cm (width × height). The images were replaced each session. The images were placed at locations 200 cm to the side of the participant. Figures 3 (robotic setting) and Fig. 4 (human agent setting) illustrate the experimental room setup.

During each session, the participants were individually brought to the room by a research assistant and were accompanied by their parents, who remained in the room throughout the entire procedure. Each trial lasted as long as the participants were comfortable in the room and ended immediately if the children indicated that they wanted to stop the interaction or if the prepared content of the interaction had been completed.

**Fig. 5:**
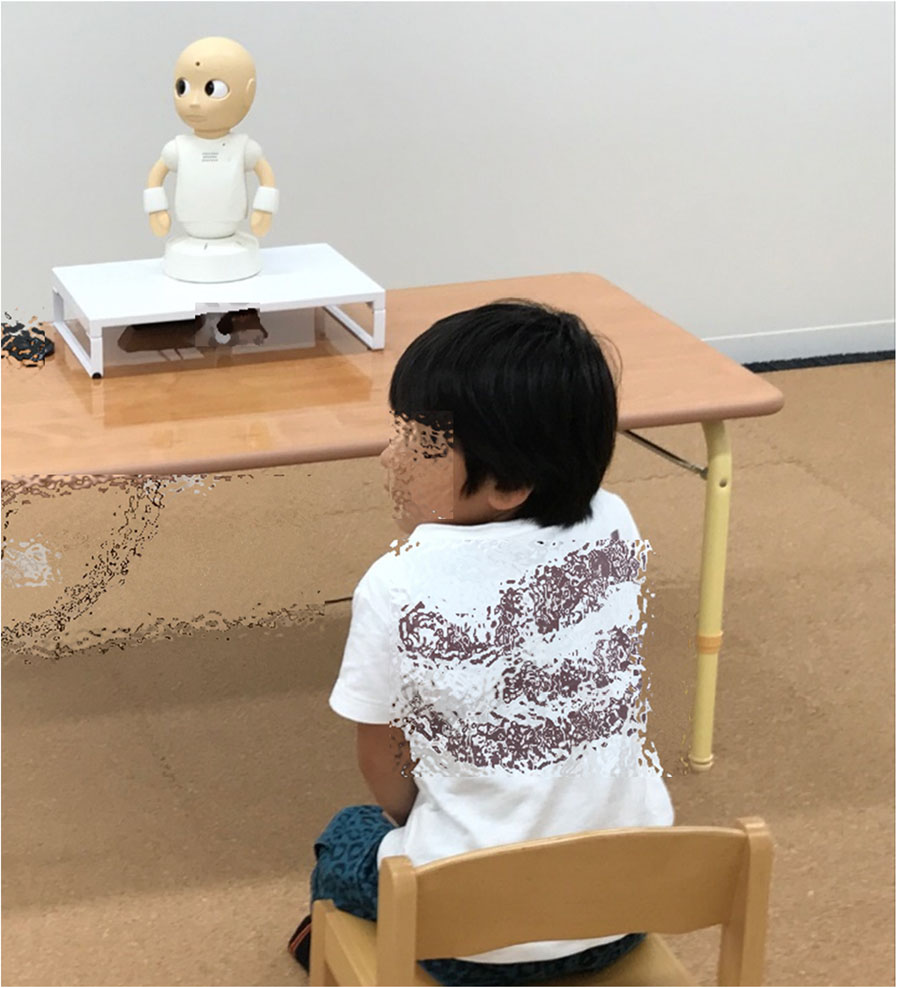
Typical interaction with the robot

During the latter half of each interaction session, after calling “Ne!,” which corresponds to the English “Hey!” (we used this syllable because /ne/ is a sentence-ending word in Japanese and conveys prosodic information [41]), the human agent or CommU attempted to induce JA by alternatively gazing toward the child for 1 s and then toward the image on the left side of the participant for 3 s; then, without calling, the agent again gazed toward the child for 1 s and then toward the image for 3 s. Then, the human agent or CommU gazed again toward the child for 1 s and then toward the other image on right side of the participant for 3 s using the same procedure, first after calling “Ne!,” and second without calling “Ne!” (i.e., the human agent or CommU attempted to induce JA four times during each interaction session). Three digital videos were set up to capture any participant response to the social prompts for an off-line analysis. Figure 5 provides an example of how the participants typically interacted with the robots. The person in this manuscript provided written informed consent to publish this picture. He agreed to publish the picture.

“Achievement of JA” was defined as a participant responding (i.e., turning to look at) to the correct target within the 3 s window. Regardless of the participant response, the human agent or robot returned to a neutral position (standing straight and facing the participant) after each prompt. Each “achievement of JA” was measured offline by counting the number of times the child turned his/her head and/or eyes in the direction of the target without fully turning, following the social prompt. Each JA event was rated a 1 (success) or 0 (failure). Each score in each interaction was calculated by simple addition (maximum score = 4). Two trainers who did not know the objective of this study independently rated the scores by watching the videotape. The raters attained a high degree of reliability (intraclass coefficient (ICC) = .98). If their scores differed, they watched the videotape together and determined the score.

### Robotic platform

CommU (Vstone Co., Ltd.) is 304 mm tall. CommU has 14 ° of freedom (DoFs) as follows: waist (2), left shoulder (2), right shoulder (2), neck (3), eyes (3), eyelids (1), and lips (1). The careful design of the eyes and multiple DoFs dedicated to controlling its field of vision contribute to its rich gaze expressions. Its face can show a range of simplified expressions that are less complex than those of a real human face. The robot’s cute shape, which resembles a child, is expected to be easy to anthropomorphize. Furthermore, its small and cute appearance is expected to help prevent fearfulness among children. In addition, CommU makes very little noise, and its interlocutor is not distressed by its noise.

### Statistical analysis

The statistical analyses were performed using SPSS version 24.0 (IBM, Armonk, NY, USA). Descriptive statistics were performed to describe the sample. The differences between the groups in terms of age, K-ABC mental processing score, and K-ABC achievement score were analyzed by performing independent samples *t* tests. The gender proportion was analyzed by performing a χ2 test. To test the first hypothesis that children with ASD would demonstrate better JA under the CommU condition than under the human agent condition, a two-way mixed ANOVA was performed to analyze the collected data from the children with ASD (JA) with one repeated factor (time; first and second interactive sessions) and one group factor (i.e., robot intervention group vs. control group). To test the second hypothesis that children with ASD would exhibit improved JA tasks with human after interacting with CommU, a two-way mixed ANOVA was performed to analyze the collected data from the children with ASD (JA) with one repeated factor (time; first and third interactive sessions) and one group factor (i.e., robot intervention group vs. control group). To test the third hypothesis that JA was facilitated differently by the human and robot agents between the children with ASD and TD children in the robotic intervention group, a two-way mixed ANOVA was used to analyze the collected data (JA) with one repeated factor (time: first and second interactive sessions) and one group factor (ASD vs. TD). An alpha level of 0.05 was employed for these analyses.

## Results

### Demographic data

Thirty children with ASD (aged 5–6 years) and 38 children with typical development (TD) (aged 5–6 years) participated in this experiment. Two children with ASD who were assigned to the control group were unable to complete the study due to distress. The ASD robotic intervention group included 16 participants (12 males), with a mean age of 70.56 ± 6.09 months. The ASD control group included 12 participants (7 males), with a mean age of 69.00 ± 4.39 months. The TD robotic intervention group included 17 participants (11 males), with a mean age of 69.88 ± 5.88 months. The TD control intervention group included 21 participants (14 males) with a mean age of 67.62 ± 6.03 months. No significant differences were observed among the groups in terms of the mean age, gender proportion, K-ABC mental processing score, or K-ABC achievement score. The SCQ total score of all participants was under 10. The participant details are presented in Table 1.

**Table 1:** Descriptive characteristics of the participants in the ASD robot intervention group, ASD control group, TD robot intervention group, and TD control group

Characteristics	ASD robot intervention group (*n* = 16) (M, SD)	ASD control group (*n* = 12) (M, SD)	TD robot intervention group (*n* = 17) (M, SD)	TD control group (*n* = 21) (M, SD)
Age in months	70.56 (6.09)	69.00 (4.39)	69.88 (5.88)	67.62 (6.03)
Sex (male:female)	12:4	7:5	11:6	14:7
K-ABC mental score	97.75 (13.42)	99.83 (17.69)	108.59 (12.54)	103.86 (13.79)
K-ABC achievement score	97.88 (19.04)	104.17 (13.29)	105.47 (13.15)	103.10 (15.79)
SCQ			1.94 (1.30)	3.33 (2.31)

*M* mean, *SD* standard deviation, *K-ABC mental* K-ABC mental processing scale, *K-ABC achievement* K-ABC achievement scale, *SRS-2*: Social Responsiveness Scale—Second Edition, T-score, *SCQ* Social Communication Questionnaire Lifetime Total Score

### Performance of the children during the JA task

**Fig. 6:**
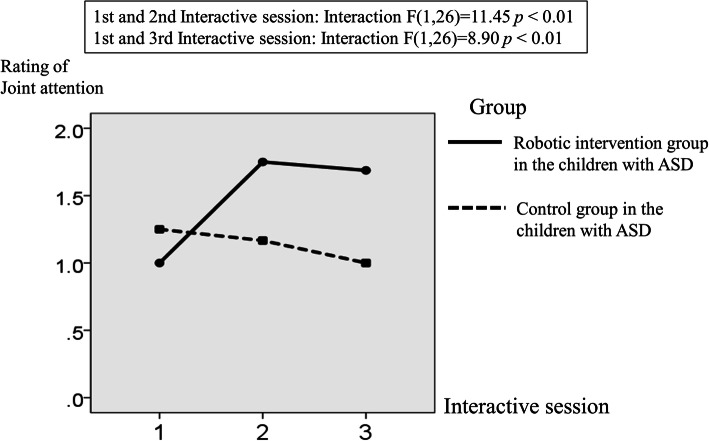
Mean rating of the joint attention in the robotic intervention and control groups (i.e., human interaction) in the children with ASD

Regarding the differences in the ratings of JA between the robotic interaction and human agent groups in the children with ASD, the results of a two-way mixed ANOVA with one repeated factor (time; first and second interactive sessions) and one group factor (i.e., robot intervention group vs. control group) showed a significant interaction between the time and group effect (F (1, 26) = 11.45; *p* < 0.01; see Fig. 6). This result supported our first hypothesis that children with ASD would demonstrate better JA under the CommU condition than under the human agent condition. In addition, the results of a two-way mixed ANOVA with one repeated factor (time; first and third interactive sessions) and one group factor (i.e., robot intervention group vs. control group) showed a significant interaction between the time and group effect (F (1, 26) = 8.90; *p* < 0.01; see Fig. 6). This result supported our second hypothesis that children with ASD would exhibit improvement in JA tasks with human after interacting with CommU.

**Fig. 7:**
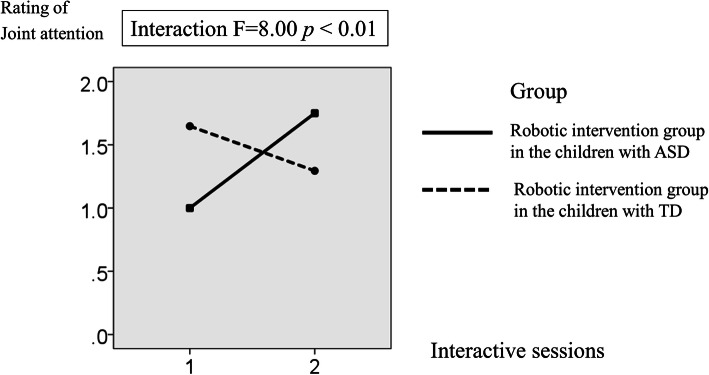
Mean rating of the joint attention in the robotic intervention group in the children with ASD and TD children

Regarding the differences in the ratings of the JA under the robotic condition between the ASD and TD children, the results of a two-way mixed ANOVA with one repeated factor (time; first and second interactive sessions) and one group factor (i.e., ASD vs. TD) showed a significant interaction between the time and group effect (F (1, 31) = 8.00; *p* < 0.01; see Fig. 7). This result supported our third hypothesis that the facilitative effect of JA due to robot intervention would be larger in children with ASD than in TD children. The details are presented in Table 2.

**Table 2:** Performance of the participants in the ASD robot intervention group, ASD control group, TD robot intervention group, and TD control group during the JA task

Group	First interaction (M, SEM)	Second interaction (M, SEM)	Third interaction (M, SEM)
ASD robot intervention group (*n* = 16)	1.00 (0.27)	1.75 (0.27)	1.69(0.29)
ASD control group (*n* = 12)	1.25 (0.31)	1.17 (0.35)	1.00 (0.33)
TD robot intervention group (*n* = 17)	1.65 (0.32)	1.29 (0.29)	1.82 (0.35)
TD control group (*n* = 21)	1.81 (0.31)	1.76 (0.22)	1.57 (0.27)

*M* mean, *SEM* standard error of the mean

Among the children with ASD in the robotic interaction group, 8 of 16 participants (50.0% of total sample) had improved JA responses, and no participants had worsened JA responses under the human agent condition before vs. after interaction with CommU (i.e., first and third interactive sessions).

## Discussion

In the current study, we examined the differences between children with ASD and TD children in their responses to induction of JA by either a human or robotic agent with clear eyes that can turn its eyes. The children with ASD who interacted with the robot had better outcomes in terms of JA than the children who interacted with a human agent during all sessions and exhibited improved performance in a JA task with human after interacting with the robot. In addition, the facilitative effect of JA due to robot intervention was larger in children with ASD than in TD children. While we used a simple design, our aim was to provide preliminary data regarding which agent better elicits JA from children with ASD and TD children with the goal of designing appropriate and tailored robotic intervention paradigms in the future.

The results of this study demonstrate that simple exposure to the robot CommU increased JA. Interestingly, this occurred in the absence of specific guidance and special settings (i.e., we used simple pictures on paper as the target objects.). Thus, utilizing this robot could contribute to improvements in JA.

Many interventions using non-social objects are available for children with ASD; however, humanoid robot-assisted interventions could be more interesting to the children than two-dimensional programs (i.e., virtual reality) [42] because the physical presence of a robot allows for a more engaging and enjoyable interaction than the use of virtual agents [43, 44]. In this study, many children with ASD showed sustained motivation to interact with the robot, which is an important factor in facilitating JA.

Madipakkam et al. [45] suggested that children with ASD exhibit an atypical response to eye contact due to their unconscious avoidance of eye contact. Thus, CommU’s clear eyes likely urged the children with ASD to pay attention to the existence of its eyes. In previous studies using Flobi, which has clear eyes and can turn its eyes, the children with ASD paid attention to the robot’s eyes [46]. In contrast, in previous studies using Nao, whose eyes are relatively small, although the children with ASD appeared to be absorbed by the robot, they could not pay attention to its eyes [14, 27, 28]. Notably, Nao is a strong attractor for children with ASD. Nao does not highly resemble a human; thus, children with ASD do not feel threatened. However, Nao’s body parts may not lead to the best results, as the attractive body parts can prevent the children from attending to a third object [29]. While brightly colored body parts attract attention, they must not be so bright as to over-stimulate the child in order to prompt JA. The color of the body parts of CommU is quiet and may contribute to the facilitation of JA in this study.

Pierno et al. [22] showed that during an imitation task, facilitation effects were only observed under the human agent condition in the TD children and only under the robot condition in ASD children. Our results are consistent with these findings in terms of the behaviors of the ASD children toward the robots and the behaviors of the TD children toward the human agents. One plausible theory might be that the complexity of the tasks completed by the robot and the human partner differ considerably. That is, variables in human behaviors include body pose, head pose, facial expression, head rotation during the experiment, and special unintentional gestures not present in the robotic experiment. The much larger number of potential uncontrolled variables in the experiments with the human partner makes it difficult to improve performance for children with ASD. Using a more complex robot like iCub [47] which has many other variables, it may be impossible to improve performance in children with ASD. Future studies using robots with different degrees of social complexity for children with various social abilities would help clarify an important factor in facilitating JA in children with ASD.

Although the changes observed in children with ASD in the robotic intervention group were statistically significant, we must consider whether they are also clinically significant. On average, the total JA score improved by 0.69 following interaction with CommU (i.e., first vs. third interactive sessions). This comprises an increase of 69% when compared to the pre-test score. In contrast, the total JA score was reduced by 0.25 in children with ASD in the control group following interaction with “human B” (i.e., first vs. third interactive sessions). This indicates that JA can improve in children with ASD in a quite limited number of sessions over a short period. Although the children in this sample demonstrated variable baseline JA skills under the human agent condition (i.e., first interactive session), 8 of 16 participants (50.0% of total sample) had improved JA responses, and no participants had worsened JA responses following interaction with CommU. Collectively, these findings suggest that robotic intervention successfully improved JA. Therefore, we believe that this increase is clinically relevant.

The strength of this study is its simple setting (i.e., we used simple pictures on paper as the target objects.) compared to that in previous studies [14, 28, 48]. The participants had no previous experience interacting with an unfamiliar robot. Notably, the children with ASD, who are generally weak in novel settings, demonstrated better JA during the interactions with the robot than with humans, and they exhibited improvement in JA tasks with human after interacting with the robot.

Certain limitations must be acknowledged. First, this study was a single session study and did not provide any indication of whether the children respond similarly over multiple sessions. Multiple sessions may offer a more extensive understanding of habituation to the robotic agent over time. While the current study did not test habituation effects in any way, it represents one of the first systematic investigations of JA using robots in children with ASD. Future studies should evaluate habituation effects with the robots by observing JA over an extended period. In addition, we do not have evidence supporting the generalizability of acquired JA to daily life. Therefore, we cannot comment on the social utility of our intervention program. The ultimate goal of the program is to enhance communication skills in daily life. In order to examine whether our program can attain this goal, future studies with a long-term longitudinal design are needed to confirm the generalized effect of this intervention in daily life (e.g., in kindergarten and at home). Third, the studied group had average cognitive skills. Clearly, future studies involving a broader range of functioning individuals are necessary to obtain a richer understanding of the potential use and impact of robotic interventions.

## Conclusions

In conclusion, as hypothesized, the children with ASD demonstrated better JA during their interaction with the robot which has clear eyes and can turn its eyes than during their interaction with the human agents. In addition, the children with ASD exhibited improved JA tasks with human after interacting with the robot. While robotic technologies are considered potential vehicles for enhancing skills in children with ASD, few studies have shown such an impact using experimental designs relevant to core challenging areas. It is both unrealistic and unlikely that robotic technology will constitute a sufficient intervention paradigm addressing all areas of impairment for all individuals with the disorder in the immediate future. Given the current state of robotic technologies, we recommend that robots be used as adjunctive tools for short-term training in individuals with ASD. The findings of this study represent a meaningful contribution to the literature on the impact of robots on JA and provide information regarding the suitability of specific robot types for therapeutic use.

## Additional file

Additional file 1: The following scripts are example of the scripts. Each session lasted approximately 5 min. (DOCX 18 kb)

## Abbreviations

ASD: Autism spectrum disorders
DSM-5: Diagnostic and Statistical Manual of Mental Disorders
JA: Joint attention
K-ABC: Kaufman Assessment Battery for Children
MINI Kids: Mini-International Neuropsychiatric Interview for Children and Adolescents
SCQ: Social Communication Questionnaire
TD: Typical development
