# Supplementary material for: The impact of robotic intervention on joint attention in children with autism spectrum disorders
Source: Mol Autism. 2018 Sep 4;9:46. doi: 10.1186/s13229-018-0230-8 (PMC6122716; doi:10.1186/s13229-018-0230-8)
Supplement: Supplementary file 1 — The following scripts are example of the scripts. Each session lasted approximately 5 min. (DOCX 18 kb) [file 13229_2018_230_MOESM1_ESM.docx]

***Supplementary Material***

One of the following four scripts was used to guide the interaction in each session. Each session lasted approximately 5 minutes.

[Script 1] Human version.

Hello!

Thanks for coming today.

You must be xxxx. Please tell me your family name.

I see! My name is yyyy. I’m from Kanazawa. Nice to meet you.

How old are you?

I see! I sometimes speak with men and women about your age.

I come here often.

Is this your first time talking with me?

I see. Are you nervous about talking to me?

I see. Are you scared of talking to me?

I would be very happy if you enjoy talking with me.

Let’s get into the rhythm of the following song now. Please get the rhythm in time to the music. Let's start!

This is a happy song! Have you ever heard of this song?

I see.

Ne! (which corresponds to the English “Hey”) (She gazes toward the animal picture on the right side. She gazes for 3 seconds.)

Do you like animals?

I see.

(She gazes toward the right side. She gazes for 3 seconds without speaking.)

I like it when animals show affection toward me. Which animals do you like best?

I see. I’m starting to want it.

I see.

Ne! (which corresponds to the English “Hey”) (She gazes toward the left side. She gazes for 3 seconds.)

Do you like juice?

I see.

(She gazes toward the left side. She gazes for 3 seconds without speaking.)

I drink apple juice every day. Which juice do you like best?

I see. I’m getting thirsty.

Let’s take a break! Good bye!

[Script 2] CommU version.

Hello!

Thanks for coming today.

You must be xxxx. Please tell me your family name.

I see! My name is CommU. I’m from Osaka. Nice to meet you.

How old are you?

I see!

I’m one year old.

I come here often.

Is this your first time talking with me?

I see. Are you nervous about talking to me?

I see. Are you scared of talking to me?

I would be very happy if you enjoy talking to me.

Let’s get into the rhythm of the following song now. Please get into the rhythm in time to the music. Let's start!

This is happy song! Have you ever heard of this song?

I see.

Ne! (which corresponds to the English “Hey”) (It gazes toward the right side. It gazes for 3 seconds.)

Do you like animals?

I see.

(It gazes toward the right side. It gazes for 3 seconds without speaking.)

I like it when animals show affection toward me. Which animals do you like best?

I see. I’m starting to want it.

I see.

Ne! (which corresponds to the English “Hey”) (It gazes toward the left side. It gazes for 3 seconds.)

Do you like juice?

I see.

(It gazes toward the left side. It gazes for 3 seconds without speaking.)

I drink apple juice every day. Which juice do you like best?

I see. I’m getting thirsty.

Let’s take a break! Good bye!
